# Data Sharing and Global Public Health: Defining What We Mean by Data

**DOI:** 10.3389/fdgth.2020.612339

**Published:** 2020-12-14

**Authors:** Nina Schwalbe, Brian Wahl, Jingyi Song, Susanna Lehtimaki

**Affiliations:** ^1^Heilbrunn Department of Population and Family Health, Columbia Mailman School of Public Health, New York, NY, United States; ^2^Spark Street Advisors, New York, NY, United States; ^3^The United Nations University - International Institute of Global Health, Kuala Lumpur, Malaysia; ^4^Department of International Health, Johns Hopkins Bloomberg School of Public Health, Baltimore, MD, United States

**Keywords:** data sharing, global public goods, public health, public health data types, global health

## Introduction

Improved information technology infrastructure and the widespread availability of mobile phones have contributed to an unprecedented amount of data produced globally each year. Data relevant to medicine and public health are being generated from a range of sources, including individuals (i.e., through social media and internet-connected devices), public and private health systems, and health researchers. These data are increasingly digitized and critical for the development of new health interventions, in particular those that use artificial intelligence and machine learning to improve health outcomes (1, 2). Recognizing that pooling and sharing data can accelerate innovation, there have been several calls in recent years to make data available as a global public good for health as part of a set of collective actions that are global in scope and required to address transnational health challenges (3, 4).

Most recently, researchers and global policy makers have called for data sharing related to the ongoing COVID-19 pandemic and developed platforms to facilitate the sharing of patient-level data for those who have contracted SARS-CoV-2 (5, 6). The pandemic has underscored the urgent need to recognize health data as a global public good with mechanisms to facilitate rapid data sharing and data governance. Doing so would support critical pandemic response activities. For example, researchers in China sequenced the SARS-CoV-2 genome and made the data publicly available through an open access platform in January 2020 (7). By making these data available, these researchers helped hasten the development of critical diagnostic assays.

Despite these efforts, there remain several persistent challenges that will need to be overcome. The technical challenges associated with data sharing have increased as datasets have grown in size and become more complex (8). There also remain several economic, legal, and political barriers that make sharing health-related data difficult. In the context of appeals for increased data sharing related to health and efforts to overcome these multifaceted challenges, different types of data and their sources are often conflated. This is problematic because the mechanisms to facilitate data sharing are often specific to data types. For example, data sharing platforms for clinical data remain in the nascent phase. While in some areas, such as cardiovascular disease, there are efforts emerging to address both drug development and clinical practice (9), in others, like genomics, where data sharing platforms are more established, the analytical software and associated outputs require significant effort for conversion and standardization (10).

Given these unique and data-specific challenges and toward establishing data as a global public good, we conducted a rapid review to characterize the current barriers to sharing data that are specific to the types of data used in public health efforts and to identify potential solutions that would support data sharing in the context of these challenges. The findings of this review could help in prioritizing the types of data that would benefit global public health and pandemic preparedness efforts through improved and expanded sharing (Table 1).

**Table 1:** Data domains, owners, and examples of data (11).

**Data domain**	**Data**	**Examples**	**Primary Owner(s)**	**Examples of existing data sharing platform**	**Types of barriers for data sharing**	**Example**
Individual	“Omics”	• Genomics • Transcriptomics • Proteomics	• Researchers • Health facilities	• Human Connectome Project • Genotype to phenotype databases (GEN2PHEN) • Nextstrain (12)	Technical (e.g., interoperability, data quality, terminology standards, dataset size, political (e.g., regulations, ownership), and economic (e.g., ownership)	Use of different analytic software and data formats is used to protect intellectual property and therefore requires significant effort for conversion and standardization
Electronic health records—structured	Demographic attributes	• Age • Gender • BMI • Location	• Private health facilities • Health provider networks • Government hospitals and networks of hospitals	• UK Clinical Practice Research Datalink (CPRD) • ESPnet (13) • FDA Mini-Sentinel (14)	Technical (e.g., interoperability, dataset size), legal (e.g., local regulations, privacy), and ethical (e.g., privacy)	Private providers may see effort required for sharing patient data not commensurate with value gained from this endeavor
	Insurance	• Plan and benefits				
	Admission/discharge	• Date • Hospital unit • Outcome (i.e., death)				
	Diagnosis	• ICD10 codes				
	Procedures	• Lab results • List of procedures conducted				
	Medications	• Prescriptions				
Electronic health records—unstructured	Documentation	• Nurse notes				
	Imaging	• Radiography • MRI				
	Audio	• Digital osculation				
Well-ness	Social media	• Tweets • Google searches	• Social media platforms	• Social media application program interface (API)	Economic (e.g., ownership), legal (e.g., local regulations), and ethical (e.g., privacy).	Companies often view sharing data as a confidential part of their business strategy
	IOT	• Heart monitor • Sleep monitor • Blood glucose device	• Device manufacturers • Individuals	• Device application program interface (API)		
Health system	Staffing	• HR database	• Private health facilities • Health provider network • Governments	• PCORnet (15)	Economic (e.g., ownership), legal (e.g., local regulations, privacy), motivational (e.g., fear of criticism) and ethical (e.g., privacy).	Insurance companies treat data as a commodity that can be monetized
	Resource utilization	• Detailed health system budgets				
	Claims, payments, and costs	• Insurance claims				
	Performance metrics	• Performance assessments • Patient satisfaction surveys				
Public health data (routine and non-routine)	Civil registration	• Death registry • Birth registry • Census	• Governments	• UK Clinical Practice Research Datalink (CPRD) • FDA Mini-Sentinel (14) • US Census (16) • UK Census (17)	Legal (e.g., local regulations, privacy), political (e.g., public trust, governance), motivational (e.g., limited value to data holders of sharing), and ethical (e.g., privacy)	Political leaders are often unwilling to share detailed or disease outbreak data for fears that it would reflect poorly on the government
	Facility level data	• HMIS		• DHIS2 (18)		
	Household surveys	• DHS • MICS		• DHS (19) • UNICEF (20)		
Health research data	Clinical trial data	• Drug trial data • Procedure trial data	• Researchers/universities • Drug manufacturers • Health research funders	• ClinicalStudyDataRequest.com • BigData@Heart (9)	Technical (e.g., interoperability), political (e.g., regulations, ownership), legal (e.g., local regulations), ethical (e.g., privacy), and economic (e.g., ownership)	Study participants might not have consented to their data being shared broadly and for purposes not specified during the consent process
	Observational study data	• Cohort study data • Case-control data	• Researchers/universities • Health research funders			

## Data Archetypes

As the volume of public health data expands and data are more interconnected, a broad range of data is being used to support research and inform global health policies and practice. We expand on an existing framework (21) to provide a classification of data into four broad archetypes (1) patient data, (2) health systems data, (3) routine public health data, and (4) health research data. It should be noted that this categorization is used to describe how these data are generated and not necessarily their application or use. This data classification is also described in further detail in Table 1.

### Patient Data

Data generated at the patient level include biomedical data (e.g., genomic data, proteomic data), electronic health records, and data generated by individuals themselves (e.g., data from wearables and social media). Sharing patient data allows for independent analysis of research results to ensure reliability, reproducibility, and accountability—all increasingly important in the context of black box algorithms based on AI. Sharing datasets can provide researchers and health intervention developers with appropriate population data references. Data sharing can therefore make an important contribution to public health by strengthening the evidence base used to make clinical and regulatory decisions (22).

### Health Systems Data

Health systems data include human resources data, service availability and utilization data (e.g., number of hospital beds, insurance claims data), and performance metrics (e.g., performance assessment results, patient satisfaction surveys). Many of these data are found in health management information systems (HMIS) datasets used around the world, including in many resource limited settings. Sharing health systems data can strengthen coordination and collaboration between the public and private sectors toward achieving common public health goals and outcomes. Sharing health systems data with the research community can also provide insights into strategies to improve the effectiveness and efficiency of health services and measuring the impact of new health policies and interventions.

### Public Health Data (Routine and Non-routine)

Routine sources include health facility and community information systems. Non-routine sources are household and other population-based surveys (e.g., demographic and health surveys, multi-indicator cluster surveys), censuses, civil registration (e.g., births and deaths) and vital statistics systems, disease surveillance systems, health facility surveys, and administrative data systems (23). Many of these data are already made available publicly either in aggregate or at the individual level. In addition to supporting research activities as already described, sharing routine and non-routine public health data can also serve to establish global health guidelines, norms, and standards. In particular, these data can be used to provide more comprehensive estimates of morbidity and mortality, including cause-specific estimates.

### Health Research Data

The data described above are largely collected for administrative purposes or for program planning and management purposes. However, a large volume of data are generated through observational and experimental health research efforts. Sharing clinical trial data can accelerate advances in public health by generating evidence on the safety and effectiveness of interventions. There are numerous examples of data from clinical trials being used to demonstrate the ineffectiveness of interventions or to improve clinical care (24). Making such data available supports transparency and reproducibility in research.

## Barriers to Sharing Data

The barriers to sharing health data have been well-characterized by Van Panhuis et al. (25) Based on our rapid review, we expanded on this nomenclature to identify the barriers specific to data sharing in global public health, and have illustrated in the table below how they might apply to each archetype.

1. Technical barriers include challenges faced by health information management systems and include lack of complete data, lost data, restrictive or conflicting data formats, lack of metadata and standards, lack of interoperability of datasets (e.g., structure or “language”), and lack of appropriate analytic solutions.
2. Motivational barriers include those that prevent individuals or organizations from readily sharing data. Specifically, these barriers include the lack of incentives, opportunity costs, fear of criticisms and disagreement on data use and access.
3. Economic barriers include both the potential and immediate costs of sharing data.
4. Political barriers are those that are inherent to the local health governance standards and typically manifest as policies and guidelines. They can also include issues of trust and ownership.
5. Legal challenges are those that arise from data collection, analysis and use, and range from concerns about who owns or controls data, transparency, informed consent, security, privacy, copyright, human rights, harm and stigma.
6. Ethical barriers include the lack of perceived reciprocity (i.e., the other party will not share data) and proportionality (i.e., deciding not to share data based on an assessment of the risks and benefits). An overarching challenge is that frameworks, laws, and regulations have not kept pace with the technological advances that are changing how data collection, analysis, sharing, or usage occur.

Data protection regulations vary across jurisdictions so definitions on ownership, control and use, as well as the tools or applications that apply these definitions need to align with applicable regulation in relevant jurisdictions (26). For transparency, when users create or generate data on a web or mobile application, the application's information collection and sharing policies are often unclear to the user. This creates a lack of transparency regarding how information may flow beyond its initial or intended audience or purpose (27). Informed consent is also problematic to implement. Collection, sharing and use of data can take place without individuals understanding it is happening or the extent that it does. This means there is a need to ensure a clear understanding on when its being collected, what is collected, how it will be shared and used, as well as how consent can be revoked (28). Security is another major barrier. Data breaches can expose confidential information, lead to third-party manipulation and violate rights or dignity. Data protection standards are needed to protect against unauthorized access, disclosure or use of personal data (29). Security is closely linked to rights and stigma, and the need to protect against the use of information to exploit, marginalize, criminalize, discriminate or exclude vulnerable or disproportionately affected populations (30).

For all data archetypes, the prospect of sharing data raises concerns related to threats to the privacy of personal information that can identify or be linked to a person (31, 32). Privacy issues are particularly acute with regard to patient data, including in resource-constrained settings where data systems are rapidly evolving, corruption is common, and data managers might lack sufficient security training (33–35). In such settings, regulations may be lacking or not be enforced (34). Even when consent procedures are in place, patients are not always explained the extent to which their data are being shared, with whom, or for what purpose (34). The use of mobile tracking apps to assist with contact tracing in the COVID-19 pandemic has highlighted many of these concerns, with multiple examples of data being sold to private companies without individual consent (11, 36, 37). Given these considerations, efforts related to data sharing in global health should require patient data and research data to be fully anonymized.

## Discussion

To date in public health, with perhaps some notable exceptions [i.e., research (38) and “omics”], there are few efforts focused on health data sharing across borders. Rather, existing data sharing activities seem to focus primarily on individual country standardization and interoperability (39). Also, where they exist, data sharing frameworks, laws, and regulations have not kept pace with the technological advances that are changing how data collection, sharing, and analysis occur (27, 30). While many of these issues are not unique to global public health, there is perhaps particular urgency in this area given the self- or group-identifying characteristics of data and the potential for misuse or harm if in the wrong hands or without proper safeguards. Addressing these pitfalls, including the misuse of public health data, will require a range of interventions bespoke to the respective data archetype and that takes into account the motivations and incentives of data owners. It will also require countries and data owners to commit to data sharing principles and acknowledge data as a global public good (4, 40).

Toward this end, there are lessons that can be learned from other disciplines where data sharing and standardization efforts are more advanced. The most well-know is the International Organization for Standardization (ISO), which has supported the development of over 23 thousand standards in a diverse range of areas, covering almost every aspect of technology and manufacturing. Less well-known is the World Meteorological Organization, which hosts a data exchange and technology transfer platform through which national weather agencies regularly share large atmospheric and meteorological data (41). The Society for Worldwide Interbank Financial Telecommunication (SWIFT) is an international cooperative based in Belgium that enables secure and standardized financial transactions around the world. The Business Identifier Codes (BIC; also known as SWIFT BIC and SWIFT codes) is a unique identification code that is used to facilitate the reliable transfer of funds between banks.

In its role as the normative global health agency, we believe that the World Health Organization (WHO) could also play an active role in coordinating data sharing efforts, engaging in partnerships and in the development of guidelines and standards, in particular for patient data, public health data, and health systems data. There are multiple examples where such efforts are already underway. WHO has developed a number of policies on data sharing (42). While legally binding, these policies do provide direction for member states (43, 44). Another is EPI-BRAIN (Epidemic Big Data Resource and Analytics Innovation Network) (45), an online platform intended to bring together public health data with information such as population movement, animal diseases meteorological, and other environmental factors to allow for analysis of large scale data sets for outbreak prediction and emergency preparedness. WHO has also recently established a new platform to support clinical characterization of patients with COVID-19 (46). By aggregating patient level data, this initiative aims to increase understanding of the severity, spectrum, and impact of the disease in the hospitalized population globally. On health systems data, WHO is collaborating with the DHIS2 platform and the University of Oslo on an opensource, web-based health management information system. While these efforts are not necessarily aggregated across countries, the architecture of the software makes this a possibility (18).

While engaging WHO does not imply compliance, by focusing on norms, standards, and guidance, including issues related to privacy, data standardization, and interoperability, WHO can serve to advance data sharing efforts for the benefit of global health.

## Author Contributions

NS conceptualized the paper. BW reviewed the literature. BW and NS jointly drafted the manuscript. JS and SL contributed to analysis and writing of the paper. NS and BW are joint first authors.

## Conflict of Interest

The authors declare that the research was conducted in the absence of any commercial or financial relationships that could be construed as a potential conflict of interest.
